# Differences between the non-steroidal aromatase inhibitors anastrozole and letrozole – of clinical importance?

**DOI:** 10.1038/bjc.2011.58

**Published:** 2011-03-01

**Authors:** J Geisler

**Affiliations:** 1 Institute of Clinical Medicine, University of Oslo, Faculty Division at Akershus University Hospital, Sykehusveien 27, Lørenskog N-1478, Norway

**Keywords:** aromatase inhibitors, breast cancer, anastrozole, letrozole, exemestane

## Abstract

Aromatase inhibition is the gold standard for treatment of early and advanced breast cancer in postmenopausal women suffering from an estrogen receptor-positive disease. The currently established group of anti-aromatase compounds comprises two reversible aromatase inhibitors (anastrozole and letrozole) and on the other hand, the irreversible aromatase inactivator exemestane. Although exemestane is the only widely used aromatase inactivator at this stage, physicians very often have to choose between either anastrozole or letrozole in general practice. These third-generation aromatase inhibitors (letrozole/Femara (Novartis Pharmaceuticals, Basel, Switzerland) and anastrozole/Arimidex (AstraZeneca, Pharmaceuticals, Macclesfield, Cheshire, UK)), have recently demonstrated superior efficacy compared with tamoxifen as initial therapy for early breast cancer improving disease-free survival. However, although anastrozole and letrozole belong to the same pharmacological class of agents (triazoles), an increasing body of evidence suggests that these aromatase inhibitors are not equipotent when given in the clinically established doses. Preclinical and clinical evidence indicates distinct pharmacological profiles. Thus, this review focuses on the differences between the non-steroidal aromatase inhibitors allowing physicians to choose between these compounds based on scientific evidence. Although we are waiting for the important results of a still ongoing head-to-head comparison in patients with early breast cancer at high risk for relapse (Femara Anastrozole Clinical Evaluation trial; ‘FACE-trial’), clinicians have to make their choices today. On the basis of available evidence summarised here and until FACE-data become available, letrozole seems to be the best choice for the majority of breast cancer patients whenever a non-steroidal aromatase inhibitor has to be chosen in a clinical setting. The background for this recommendation is discussed in the following chapters.

Adjuvant endocrine therapy has an important role in postmenopausal women (PMW) with hormone receptor-positive (HR+) breast cancer. Efficacy of anti-hormonal treatment of early breast cancer is based on the fact that estrogens may stimulate the growth of residual cancer cells or contribute to the initiation of a new primary cancer over time.

Selective estrogen-receptor (ER) modulators, such as tamoxifen, have been the gold standard of care for women with HR+ breast cancer for the last 30 years (Jordan, 2004; Geisler *et al*, 2008). Tamoxifen therapy for 5 years can reduce the odds of recurrence and death by 47 and 26%, respectively (Early Breast Cancer Trialists' Collaborative Group, 2005; Howell *et al*/ATAC Trialists Group, 2005).

Aromatase inhibitors (AIs) have now replaced tamoxifen as the standard of care for adjuvant endocrine therapy in the treatment of PMW with hormone-sensitive breast cancer. Three generations of AIs have been developed during the last 3 decades. Of these, the third-generation AIs have more favorable tolerability profiles and are more selective and/or potent compared with first- and second-generation agents (Nabholtz *et al*, 2000; Mouridsen *et al*, 2001). There are two broad categories of third-generation AIs (Lønning and Geisler, 2008). The reversible non-steroidal agents include anastrozole and letrozole (triazole derivatives). The third agent, exemestane, is an androstenedione derivative that functions as an irreversible steroidal inhibitor (or inactivator). The triazole derivatives bind to the cytochrome P-450 component of the aromatase enzyme, whereas the steroidal compound exemestane binds to the substrate-binding pocket of the aromatase enzyme (Geisler *et al*, 1998), leading to its degradation (Figure 1). AIs are now widely used as first-line therapy for PMW with hormone-sensitive early breast cancer, as first-line therapy for metastatic disease, and as second-line agents in cases of tamoxifen resistance. This review emphasises the potency and emerging efficacy differences between third-generation AIs and places particular emphasis upon comparisons between anastrozole and letrozole.

## Differences in the mechanism and potency of AI-induced estrogen suppression

### *In vitro* results

Several studies evaluating the reduction of aromatisation *in vitro* have compared the potency of third-generation AIs (Bhatnagar *et al*, 2001). Miller (1999) used two *ex-vivo* assays of aromatase activity in particular fractions of breast cancer tissue and in mammary fibroblast cell cultures. Aromatase activity was effectively inhibited in both particular fractions of breast cancers and cultures of mammary adipose tissue fibroblasts. In another study by Miller *et al* (2001), immunohistochemical analyses revealed that treatment with anastrozole or letrozole resulted in significant decreases in progesterone receptor (PgR) expression, a marker for estrogen function. Bhatnagar *et al* (2001) demonstrated that in rodent cells, normal human adipose fibroblasts, and human cancer cell lines, letrozole was consistently 10–30 times more potent than anastrozole in its ability to inhibit intracellular aromatase. It is important to note, however, that *in vitro* assays may not accurately reflect the degree of inhibition produced/achieved *in vivo*.

### Animal models

An intratumoural aromatase model system in mice was developed to mimic postmenopausal ER+ breast cancer. This animal model takes into account the importance of locally produced (intratumoural) aromatase, as well as the fact that breast cancer occurs mainly in PMW. Although these mice (ovariectomised, athymic, and immunosuppressed nude mice) have no significant peripheral estrogen production capability and no adrenal androgen production, AI efficacy is assessed after inoculation with human breast carcinoma cells transfected with the human aromatase gene (MCF-7_arom_ cells) (Brodie *et al*, 2005). Using this aromatase xenograft model, letrozole was shown to be more effective than tamoxifen in suppressing breast tumour growth without causing endometrial proliferation. Additionally, tumours continuing to progress on tamoxifen therapy remained sensitive to second-line therapy with letrozole.

### *In vivo* measurements

The biochemical efficacy of AIs *in vivo* may be determined from their effects on total body aromatisation, as well as from changes in plasma and tissue estrogen levels. Because of their high sensitivity, tracer methods that allow the calculation of whole-body aromatase inhibition are preferred (Lønning and Geisler, 2008). Unfortunately, these methods are labor-intensive, and analyses are usually limited to small numbers of patients. Plasma estrogen measurement is a cruder but simpler method that allows screening of much larger numbers of patients. As there may be significant variation between local estrogen synthesis in addition to uptake of estrogens from the circulation in some tumours, direct measurement of intratumour estrogens is required to assess the potency of AI estrogen suppression in malignant target tissues (Lønning and Geisler, 2008).

The third-generation AIs approved by the Food and Drug Administration (anastrozole, letrozole, and exemestane) are highly selective competitive inhibitors/inactivators of the aromatase enzyme. Although first- and second-generation AIs inhibit estrogen synthesis *in vivo* up to 90%, third-generation compounds reproducibly cause ⩾98% aromatase inhibition in humans (Geisler *et al*, 2008). Table 1 provides a comparison of the total body aromatase inhibition of third-generation AIs compared with the first- and second-generation compounds. Furthermore, suppression of plasma levels of estrogens by >90% has been consistently demonstrated with all third-generation AIs (Dowsett *et al*, 1995; Geisler *et al*, 1996, 1998, 2002, 2008).

**Figure 2:**
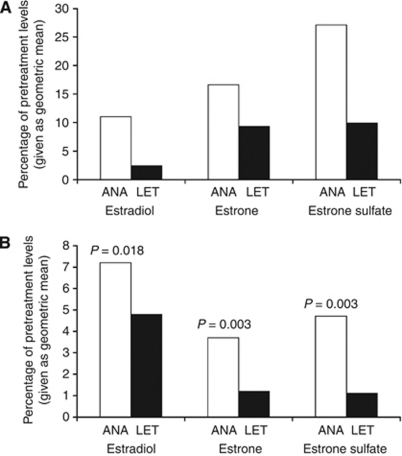
(**A** and **B**) Influence of anastrozole (ANA) *vs* letrozole (LET) on tissue (**A**) and plasma (**B**) estrogen levels: percentage of pretreatment levels (given as geometric mean) (Geisler *et al*, 2001, 2008).

Among third-generation AIs, letrozole seems to produce the most extensive estrogen suppression. Results from an intrapatient crossover study revealed that letrozole (2.5 mg daily) consistently resulted in more potent aromatase inhibition compared with 1.0 mg anastrozole (Geisler *et al*, 2002). In PMW undergoing primary treatment for locally advanced ER+/PgR+ breast cancer, letrozole suppressed pretreatment tumour levels of estradiol (E2), estrone (E1), and estrone sulfate (E1S) by 97.6, 90.7, and 90.1%, respectively (Figure 2A) (Geisler *et al*, 2008). This level of suppression is superior to that previously reported for anastrozole using the same methods: 89.0, 83.4, and 72.9%, respectively (Geisler *et al*, 2001). Direct comparisons of reanalyzed samples also found superior suppression of plasma estrogen levels with letrozole compared with anastrozole (Geisler *et al*, 2008), E2 (average suppression by 95.2 *vs* 92.8% *P*=0.018), E1 (98.8% suppression *vs* 96.3% *P*=0.003), and E1S (98.9% suppression *vs* 95.3% *P*=0.003) (Figure 2B). Recently, Dixon *et al* (2008) confirmed that letrozole reduces plasma estrogen levels to a greater degree than does anastrozole at clinical doses. The results of these two translational studies, Geisler *et al* (2008) and Dixon *et al* (2008), raise the question of whether differences in potency translate into differences of clinical importance. Although it has been postulated by some authors that aromatase inhibition above a defined level (f. eks. 90% inhibition) might not increase the clinical efficacy, the lessons we learned from clinical studies through the last 3 decades suggest that estrogen suppression and clinical efficacy are tightly correlated also above 90% aromatase inhibition *in vivo*. One example is the head-to-head comparison of aminoglutethimide (aromatase inhibition: 90%) and letrozole (aromatase inhibition: >99%) showing clearly superiority of letrozole in the setting of advanced breast cancer (Gershanovich *et al*, 1998). However, because of the very different side-effect profiles of aminoglutethimide and letrozole when given in the clinically established doses, with the latter being much less toxic, an influence of compliance problems during aminoglutethimide treatment on the study results cannot be excluded.

It is still a matter of discussion whether differences between 95 and 99% inhibition of aromatisation *in vivo* translate into significant different clinical response rates and times. In addition, although all major phase III trials performed with anastrozole and letrozole used the same doses of the individual drugs (1 mg anastrozole once daily (o.d.) and 2.5 mg letrozole o.d.), it might be questioned that the optimal clinical dose has been used - at least for anastrozole. Although a direct intrapatient crossover trial with anastrozole 1 mg o.d. *vs* 10 mg o.d. did not reveal a significant difference between the two daily doses, it is important to mention that 8 out of 10 patients experienced a better aromatase inhibition while on the 10 mg o.d. dose (Geisler *et al*, 1996). In contrast, the corresponding study comparing letrozole 0.5 mg o.d with 2.5 mg o.d. did not reveal a major difference between the two tested doses (Dowsett *et al*, 1995). In conclusion, a suboptimal dosage of anastrozole cannot be totally ruled out. However, this problem cannot be solved by simply increasing the daily dose of anastrozole. This would require to repeat all phase III trials with the higher dose of anastrozole to evaluate clinical responses and even more important that is, side effects. With patents expiring for all aromatase inhibitors in a short-time frame, this is probably not of any interest for the involved pharmaceutical company.

### Clinical studies

#### Neoadjuvant setting.

Letrozole is the only AI that has been demonstrated to possess significantly superior efficacy to tamoxifen in the neoadjuvant setting, and is the only AI to have received approval from several countries for use in this setting. In a randomised, double-blind study in PMW (*N*=337) with ER+ and/or PgR+ breast cancer comparing letrozole with tamoxifen (Eiermann *et al*, 2001), letrozole was superior to tamoxifen in overall objective response rate (55 *vs* 36% *P*<0.001). Furthermore, breast-conserving surgery (BCS) was possible in more patients treated with letrozole (45 *vs* 35% *P*=0.022). In another double-blind study of PMW with ER+ and/or PgR+ breast cancer ineligible for BCS, patients were randomly assigned to receive either 4 months of neoadjuvant letrozole or tamoxifen (Ellis *et al*, 2001). Letrozole had a significantly better response rate (60 *vs* 41% *P*=0.004), and letrozole-treated patients had significantly more BCS (48 *vs* 36% *P*=0.036). Differences in response rates were most marked for patients with human epidermal growth factor receptor (HER) 1/2+ tumours (88 *vs* 21% *P*=0.0004).

The Pre-operative ‘Arimidex’ Compared To Tamoxifen Trial was a randomised, multicenter study comparing anastrozole (*n*=228) with tamoxifen (*n*=223) as neoadjuvant treatment in PMW with HR+, large, operable breast cancer (Cataliotti *et al*, 2006). Objective responses for anastrozole and tamoxifen occurred in 39.5 and 35.4% of patients, respectively (ultrasound), and 50.0 and 46.2% of patients, respectively (caliper). In the intent-to-treat (ITT) population, surgery became feasible after 3 months of hormonal therapy in 38.1 *vs* 29.9% of anastrozole- and tamoxifen-treated patients, respectively (*P*=0.11). In patients receiving endocrine therapy only (*n*=314), surgery became feasible in 43% of patients receiving anastrozole *vs* 30.8% receiving tamoxifen after 3 months of treatment (*P*=0.04).

In contrast to these findings, the Immediate Preoperative Anastrozole, Tamoxifen, or Combined With Tamoxifen trial did not find any significant benefit with anastrozole compared with tamoxifen. Postmenopausal women with ER+ invasive breast cancer were randomised to one of three treatment groups: neoadjuvant anastrozole (*n*=113), tamoxifen (*n*=108), or anastrozole plus tamoxifen (*n*=109) (Smith *et al*, 2005). There were no significant differences in clinical objective response rates (caliper) across treatment groups (*P*=0.87; anastrozole *vs* tamoxifen).

The outcome of patients in need of neoadjuvant therapy may also be altered by the frequency of complete pathological responses during therapy with an AI. Although complete pathological responses have been observed in a study published by Dixon *et al*, these were not found in the corresponding group of patients treated with anastrozole (Dixon *et al*, 1999).

### Initial adjuvant setting

#### Early Breast Cancer

##### Arimidex, Tamoxifen, Alone or in Combination (ATAC) trial

This phase III multinational, randomised, double-blind trial initially comprised 9366 PMW with breast cancer (Baum *et al*, 2002). It compared anastrozole alone or in combination with tamoxifen with tamoxifen monotherapy following breast cancer surgery. Combination therapy was not significantly more beneficial than tamoxifen alone, so the combination arm was terminated early, leaving further analyses limited to 6241 patients (Baum *et al*, 2002). At a median follow-up of 68 months, treatment with anastrozole in HR+ patients revealed a significant benefit in disease-free survival (DFS) (hazard ratio (HR)=0.83; 95% confidence interval (CI) 0.73–0.94; *P*=0.005) and time to recurrence (TTR) (HR=0.74; 95% CI: 0.64–0.87; *P*=0.0002). However, there was no significant improvement in time to distant recurrence (TTDR; HR=0.84; 95% CI: 0.70–1.00; *P*=0.06) or overall survival (OS; HR=0.97) in the relevant HR+ patient population (Howell *et al*, 2005). At 100 months of follow-up, investigators observed a significant benefit in TTDR (risk of distant metastasis (DM)) in HR+ patients receiving anastrozole (HR=0.84; 95% CI: 0.72–0.97, *P*=0.022). Anastrozole also continued to provide a significant benefit in DFS (HR=0.85; 95% CI: 0.76–0.94; *P*=0.003) and TTR (HR=0.76; 95% CI: 0.67–0.87; *P*=0.0001). Although there were improvements in DFS, there was still no OS benefit for anastrozole *vs* tamoxifen (472 *vs* 477 events, respectively; HR=0.97; 95% CI: 0.86–1.11; *P*=0.7) (Forbes *et al*, 2008). The key efficacy endpoints of the ATAC trial across treatment settings are summarised in Table 2.

##### Breast International Group (BIG) 1–98 trial

This large international phase III, randomised, double-blind trial was independently run by the International Breast Cancer Study Group (IBCSG), a cooperative academic group interested in an analysis of letrozole *vs* tamoxifen in PMW with HR+ breast cancer (ITT: *N*=8010; two-arm option: tamoxifen, *n*=911, letrozole, *n*=917; four-arm option: tamoxifen, *n*=1548, letrozole, *n*=1546, tamoxifen followed by letrozole, *n*=1548, letrozole followed by tamoxifen, *n*=1540) (Thürlimann *et al*, 2005). The key efficacy endpoints across treatment settings are summarised in Table 2.

Initial results (*N*=8010) at a median follow-up of 25.8 months demonstrated the superiority of letrozole over tamoxifen in prolonging DFS and TTR (but not OS). Letrozole-treated patients had a significant early benefit in TTDR, with a 27% reduction in risk for DM at 25.8 months' median follow up (Thürlimann *et al*, 2005). A retrospective study (*N*=7707) of BIG 1–98 confirmed these results; there was a pronounced 30% reduction in early DM (at 2 years) with letrozole compared with tamoxifen (87 *vs* 125 events, respectively) (Mauriac *et al*, 2007). Based on the superiority of letrozole *vs* tamoxifen at 25.8 months, the IBCSG decided to unblind the tamoxifen monotherapy arm only and to allow patients the choice to crossover to letrozole therapy; 619 (25.2%) made the informed choice (with counseling) to crossover to the letrozole arm. As a result of the crossover, in addition to the ITT analysis, a censored analysis was performed to account for crossover patients (Mouridsen *et al*, 2009). Analysis of ITT patients at a median follow-up of 76 months of letrozole or tamoxifen monotherapy demonstrated a continued significant benefit in DFS (HR=0.88; 95% CI: 0.78–0.99; *P*=0.03) and TTDR (HR=0.85; 95% CI: 0.72–1.00; *P*=0.05) with letrozole (Mouridsen *et al*, 2009). There were 40 fewer deaths with letrozole, demonstrating an emerging survival benefit (303 *vs* 343; HR=0.87; 95% CI: 0.75–1.02; *P*=0.08). In the censored analysis, accounting for patients who crossed-over to letrozole, letrozole-treated patients had benefit in DFS (HR=0.84; 95% CI: 0.74–0.99), TTDR (HR=0.81; 95% CI: 0.68–0.96), and OS (HR=0.81; 95% CI: 0.69–0.94). However, the IBCSG concluded that early crossover to letrozole possibly biased the ITT analysis in favour of tamoxifen and the censored analysis in favour of letrozole, making accurate assessments of OS difficult. To adjust for this potential bias, an additional inverse probability of censoring weighted analysis was carried out to provide a more accurate estimate of the clinical benefit of letrozole. The results demonstrated that 5 years of letrozole significantly improved DFS by 15% (HR=0.85; 95% CI: 0.76–0.96) and OS by 17% (HR=0.83; 95% CI: 0.7–0.97; *P*<0.05) (Giobbie-Hurder *et al*, 2009), reflecting the statistics that might have been observed in the absence of selective crossover (Mouridsen *et al*, 2009). It is important to note that with the emergence of significantly improved treatments, the issue of crossover is being addressed in many trials and is not unique to this trial.

## Implications of CYP2D6 genotype

The potential effect of CYP2D6 genetic variants on clinical response in tamoxifen-treated breast cancer patients has recently gained much interest. CYP2D6 is predominantly responsible for the 4-hydroxylation of tamoxifen leading to its most active metabolites, 4-hydroxytamoxifen and endoxifen (Dehal and Kupfer, 1997). CYP2D6 poor metabolisers have been reported to be at higher risk for recurrence compared with CYP2D6 wild-type patients (Schroth *et al*, 2009). Although the data are still somewhat conflicting at this point (Dezentjé *et al*, 2009), CYP2D6 genotyping might become the first predictive factor in breast cancer patients that may be analysed in a blood sample in the near future. The reported differences between the tamoxifen arms and the AI-arms in the large clinical phase III studies in early breast cancer have to be re-evaluated in the light of these novel findings.

## Influence on distant metastasis

Indirect information from the ATAC and BIG 1–98 trials indicates that differences in clinical efficacy exist between anastrozole and letrozole in the initial adjuvant setting. Both AIs improve DFS compared with tamoxifen in patients with HR+ disease. The most common type of recurrence seen 2 to 3 years post surgery is DM, a well-recognised predictor of breast cancer survival (Saphner *et al*, 1996; Mansell *et al*, 2009). Only letrozole has been shown to significantly reduce early DM events (30% reduction in the risk of DM at 2 years) (Mauriac *et al*, 2007). A significant reduction in DM events was also seen with anastrozole in HR+ patients but only at 100 months of follow-up (Forbes *et al*, 2008). In contrast, exploratory analysis of the ATAC trial confirmed that most of the early benefit with anastrozole was not in the prevention of distant disease; at 2.5 years, there was a 7% benefit in the prevention of DM (Houghton, 2006).

## Novel antiestrogens *vs* aromatase inhibitors

The development of fulvestrant (Faslodex, AstraZeneca, Pharmaceuticals, Macclesfield, Cheshire, UK), a unique ER antagonist and downregulator has caused considerable attention. Although experience with fulvestrant in early breast cancer is limited at this point, recent head-to-head comparisons in the metastatic setting have revealed that fulvestrant (500 mg intramuscular (i.m.)/4 w) is at least as effective as anastrozole (1 mg oral (p.o.)/o.d.) with significantly improved time to progression for patients in the fulvestrant arm (Robertson *et al*, 2009). Thus, the novel increased dosage of fulvestrant is a promising endocrine treatment option in all settings of breast cancer, and data for patients with early breast cancer are awaited in a short-time frame.

## The Femara anastrozole clinical evaluation trial (FACE)

Although the ATAC and BIG 1–98 trials have provided an extensive data set for anastrozole and letrozole in early breast cancer, no head-to-head trial of these two AIs has been conducted in this setting. The ongoing FACE trial was designed to prospectively address potential efficacy and safety differences (O'Shaughnessy, 2007). This phase III open-label, randomised, multicenter study includes node-positive patients randomised to receive either early adjuvant letrozole or anastrozole. The primary objective is to compare DFS at 5 years. Secondary objectives are to assess safety, OS, time to DM, and time to contralateral breast cancer. The trial was designed to differentiate between the two drugs in the shortest possible time by enrolling patients at increased risk of early recurrence of breast cancer, so that the number of events required to initiate analysis will be obtained more quickly. The results may help refine treatment strategies for PMW with breast cancer.

## Adverse events (AE) of AI therapy

Because of the extended duration of adjuvant endocrine therapy, patient tolerability issues and their potential influence on compliance and therapeutic outcome are important themes. All in all, third-generation AIs seem to have very similar toxicity profiles. Most side effects are explained by the general estrogen deprivation, predictable and similar to those of natural menopause. They may include hot flashes, arthralgia, osteoporosis, fractures, hypercholesterolemia, and cardiovascular events (see Table 3 for an overview). Side effects like thromboembolic events and endometrial cancer that are well established for antiestrogen therapy, are rarely seen during therapy with aromatase inhibitors. Although novel antiestrogens like raloxifene are less toxic compared with tamoxifen when tested head-to-head in women at increased risk for development of breast cancer, raloxifene has recently been shown to be less effective in preventing breast cancer, too (Vogel *et al*, 2010). Although adverse events of third-generation AIs have been reviewed extensively in the available literature (Thürlimann *et al*, 2005; Buzdar *et al*, 2006; Coates *et al*, 2007; Coombes *et al*, 2007), this issue will only be briefly discussed here.

### Lipid metabolism and cardiovascular symptoms

Blood cholesterol was comprehensively and systematically measured in the BIG 1–98 trial under non-fasting conditions every 6 months (Thürlimann *et al*, 2005). At a mean follow-up of 25.8 months, there was more hypercholesterolemia in the letrozole arm (43.6, *vs* 19.2% in the tamoxifen arm), but this AE was predominantly low grade. Importantly, median changes in cholesterol levels remained stable at 6, 12, and 24 months (0, 0, and −1.8%) in the letrozole group but decreased in the tamoxifen group at each assessment (−12.0, −13.5, and −14.1%). These results support a greater lipid-lowering effect of tamoxifen rather than a detrimental effect of letrozole. Safety analysis at a median of 60.5 months confirmed earlier results. Although there was an increased lipid impairment *vs* tamoxifen, hypercholesterolemia was predominantly of low grade (Thürlimann *et al*, 2009).

In contrast, hypercholesterolemia was not a predefined AE in the ATAC trial; however, results indicated a significantly greater rate of hypercholesterolemia in the anastrozole arm than in the tamoxifen arm (9 *vs* 3% odds ratio 2.73; *P*<0.0001) (Buzdar *et al*, 2006). In conclusion, blood lipids seem to be slightly and equally increased during therapy with all third-generation AIs (anastrozole, letrozole, and exemestane) when compared directly with tamoxifen, because of the lipid-lowering effects of tamoxifen and not because of a lipid-increasing effect of AIs.

### Musculoskeletal symptoms and bone loss

Up to 30% of all women taking AIs report myalgia or arthralgia. In the adjuvant setting, the rate of muculoskeletal symptoms is significantly higher for all third-generation AIs compared with tamoxifen. These events generally emerge early in treatment, are low grade and improve with time.

In addition, AIs cause a significant increase in both bone resorption and formation. Osteoporosis and increased fracture rates occur in some patients when using AIs (Table 3). Although preclinical studies suggested that bone loss may be less during treatment with a steroidal AI (exemestane) compared with non-steroidal AIs (anastrozole and letrozole), there is no evidence at all from clinical trials confirming these hypotheses. In contrast, a randomised trial of healthy volunteers demonstrated that all AIs have a similar effect on burn turnover (McCloskey *et al*, 2006). Because of the early screening for osteopenia and osteoporosis whenever AIs are implemented in patients with early breast cancer and liberal use of calcium, vitamin D, and bisphosphonates, the issue of bone loss seems to be solved for the majority of patients.

### Endocrine resistance and future research

Acquired, as well as *de novo*, resistance to aromatase inhibition remains a major concern in clinical practice. Future research will seek to improve our understanding of how to treat AI-resistant breast cancers and, perhaps more importantly, how to prevent the onset of AI-induced resistance. Studies in model systems have suggested that tumour cells gradually adapt to low estrogen levels during AI-treatment, eventually acquiring resistance. However, early microarray profiling data suggest extensive heterogeneity in the resistance mechanisms involved. When estrogen levels are profoundly suppressed, *in vitro* models of *de novo* resistance suggest that tumour cells may have the capability to develop estrogen hypersensitivity through changes in gene expression and regulation of growth factor signalling pathways (Santen *et al*, 2005). On the basis of these findings, aromatase inhibitors and anti-HER-2 agents (trastuzumab, lapatinib) have been tested in combination in clinical studies showing improved progression-free survival in the combination arms (Johnston *et al*, 2009; Kaufman *et al*, 2009). Interestingly, recent data suggest also a complex recruitment of nuclear receptor co-activators/-suppressors to the ER during AI treatment *in vivo* (Flågeng *et al*, 2009). Thus, ER-cofactors have become an area of intense research aiming to develop novel drugs that may interfere with the ER–cofactor complexes. Other determinants of endocrine resistance in breast cancer patients have recently been reviewed by Musgrove and Sutherland (2009).

The development of novel aromatase antibodies like antibody 677 may provide novel tools for the identification of PMW with ER+ and aromatase-positive tumours that will respond to AI therapy before it is initiated (Geisler *et al*, 2010).

## Conclusions

Aromatase inhibition is now established as standard care in both early and metastatic breast cancer for HR+ PMW following crucial phase III trials involving >30 000 patients. Highest *in-vivo* potency, with superior E2 suppression in human breast cancer tissue, has been demonstrated with letrozole compared with anastrozole. In addition to being the most potent non-steroidal AI, letrozole is the only AI that has demonstrated superior efficacy in both the neoadjuvant and adjuvant settings compared with tamoxifen. A significant reduction of early DM in the adjuvant setting has been shown with letrozole, and a survival benefit is emerging with longer follow-up in the BIG 1–98 trial. In contrast, 100-month survival data from the ATAC trial do not demonstrate an OS benefit for anastrozole *vs* tamoxifen. Third-generation AIs have a predictable and manageable safety profile, with AEs similar to symptoms of menopause. The severe AEs associated with tamoxifen are not observed with AIs. Direct head-to-head comparisons of AIs are needed to further elucidate differences among third-generation compounds. Until such data are available, the clinical data at hand suggest that the third-generation aromatase inhibitor letrozole may be the best choice when a non-steroidal AI is indicated.

Finally, whenever tamoxifen is used in clinical trials in the future, CYP2D6 genotyping should be implemented to further clarify the role of this potentially predictive marker.

## Figures and Tables

**Figure 1:**
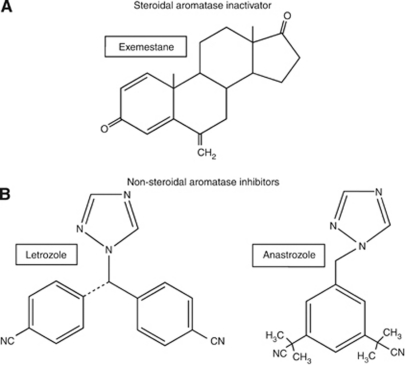
Chemical structures of currently used antiaromatase compounds. (**A**) Steroidal aromatase inactivator. (**B**) Non-steroidal aromatase inhibitors.

**Table 1:** Inhibition of whole-body aromatisation among three generations of aromatase inhibitors

**Generation**	**Compound**	**Dose (mg)**	**% Inhibition**	**Reference**
First	Aminoglutethimide	250 qid	90.6	MacNeill *et al* (1992)
	Formestane (IM)	250 2w	84.8	Jones *et al* (1992)
Second		500 2w	91.9	
		500 w	92.5	
	Formestane (po)	125 od	72.3	MacNeill *et al* (1992)
		125 bid	70.0	
		250 od	57.3	
Second	Rogletimide	200 bid	50.6	MacNeill *et al* (1992)
		400 bid	63.5	
		800 bid	73.8	
Second	Fadrozole	1 bid	82.4	Lønning *et al* (1991)
		2 bid	92.6	
Third	Anastrozole	1 od	96.7	Geisler *et al* (1996)
		1 od	97.3	Geisler *et al* (2002) a
Third	Letrozole	2.5 od	>98.9	Dowsett *et al* (1995)
		2.5 od	>99.1	Geisler *et al* (2002) a
Third	Exemestane	25 od	97.9	Geisler *et al* (1998)

Abbreviations: od=once daily; bid=twice daily; qid=four times daily; w=weekly; 2w=twice weekly; po=oral; IM=intramuscular.

a Detected in a direct, intrapatient crossover study.

**Table 2:** Key efficacy endpoints of major clinical trials across treatment settings

	**ATAC^a^**		**BIG 1-98^b^**			
**Follow-up (months)**	**68**	**100**	**25.8**	**76**	**76 Censored^c^**	**76 IPCW^c^,d**
DFS	0.83 (*P*=0.005)	0.85 (*P*=0.003)	0.81 (*P*=0.003)	0.88 (*P*=0.03)	0.84	0.85
TTR	0.74 (*P*=0.0002)	0.76 (*P*=0.0001)	0.72 (*P*<0.001)	NR	NR	NR
TTDR	0.84 (*P*=0.06)	0.84 (*P*=0.022)	0.73 (*P*=0.001)	0.85 (*P*=0.05)	0.81	NR
OS	0.97 (*P*=0.7)	0.97 (*P*=0.7)	0.86 (*P*=0.16)	0.87 (*P*=0.08)	0.81	0.83
Reference	Howell *et al* (2005)	Forbes *et al* (2008)	Thürlimann *et al* (2005)	Mouridsen *et al* (2009)	Mouridsen *et al* (2009)	Mouridsen *et al* (2009)

Abbreviations: ATAC=arimidex, tamoxifen, alone or in combination; BIG=breast international group; IPCW=inverse probability of censoring weighted analysis; NR=not reported; DFS=disease-free survival; TTR=time to recurrence; TTDR=time to distant recurrence; OS=overall survival

a Hormone receptor-positive population.

b Intent-to-treat population.

c Follow-up censored at selective crossover.

d The weighting adjusts for factors associated with OS and with selective crossover, including baseline factors such as age, nodal status, tumour grade, and time varying performance status.

**Table 3:** Incidence (%) of third-generation aromatase inhibitor-related adverse events compared with tamoxifen

	**Anastrozole**	**Letrozole**
Study	ATAC	BIG 1–98
*N*	6186	8010
Follow-up (mos)	68	60.5
Arthralgia	35.6 *vs* 29.4	21.9 *vs* 16.5
	*P*<0.0001	NR
Osteoporosis	NR	NR
Fractures	11.0 *vs* 7.7	7.5 *vs* 5.7
	*P*<0.0001	NR
Hot flashes	35.7 *vs* 40.9	35.2 *vs* 39.5
	*P*<0.0001	NR
Hypercholesterolemia	NR	48.7 *vs* 24.1
		NR
Overall cardiac events	NR	5.6 *vs* 5.4
		NR
Ischemic cardiovascular disease	4.1 *vs* 3.4	2.1 *vs* 1.7
	*P*=0.1	NR
Reference	Howell *et al* (2005)	Thürlimann *et al* (2009)

Abbreviations: ATAC=arimidex, tamoxifen, alone or in combination; BIG=breast international group; NR=not reported; NS=not significant.
